# Camera Port Swapping in Transperitoneal Robotic Partial Nephrectomy: A Feasible Alternative to the Retroperitoneal Approach for Posterior Renal Tumors

**DOI:** 10.3390/jcm14176109

**Published:** 2025-08-29

**Authors:** Jinhyung Jeon, Sungun Bang, Jeong Hyun Lee, Jong Kyou Kwon, Do Kyung Kim, Kang Su Cho

**Affiliations:** 1 Department of Urology, Yongin Severance Hospital, Yonsei University College of Medicine, Yongin 16995, Republic of Korea; jun1644@yuhs.ac; 2 Department of Urology, Prostate Cancer Center, Gangnam Severance Hospital, Yonsei University College of Medicine, Seoul 06229, Republic of Korea; bbsungun@yuhs.ac (S.B.); spiralcloud@yuhs.ac (J.H.L.); jkstorm@yuhs.ac (J.K.K.); dokyung80@yuhs.ac (D.K.K.); 3 Center of Evidence Based Medicine, Institute of Convergence Science, Yonsei University, Seoul 03722, Republic of Korea

**Keywords:** robotic surgical procedures, nephrectomy, kidney neoplasms

## Abstract

**Background**: Robotic partial nephrectomy (RPN) for posterior renal tumors can be performed via the transperitoneal approach (TA); however, it may provide suboptimal visualization of posterior lesions compared to the retroperitoneal approach (RA). The camera port swapping (CPS) technique was developed to enhance intraoperative visualization and robotic arm maneuverability during TA-RPN. **Methods**: We conducted a retrospective review of patients who underwent RPN for posterior renal tumors between 2018 and 2024 using either TA with the CPS technique (*n* = 35) or RA (*n* = 29). All procedures used the da Vinci Xi surgical system, and the CPS technique involved repositioning the camera port intraoperatively when standard visualization proved inadequate during TA. Propensity score matching (1:1) was performed based on tumor size and body mass index to compare outcomes (*n* = 21 in each group). **Results**: Propensity score-matching analysis revealed that body mass index, tumor size, and RENAL nephrometry score were comparable between the two groups. The positive surgical margin was zero in all patients. The warm ischemia time was 22 min (0–44 min) in the TA-CPS group and 18 min (7–45 min) in the RA group (*p* = 0.504). No complications of Clavien–Dindo classification grade > 3 occurred in the TA-CPS group, while one occurred in the RA group (*p* = 1.000). Renal function decline was 4.8% in the TA-CPS group and 19% in the RA group (*p* = 0.343). Trifecta achievement rates were also comparable: 95.2% in the TA-CPS group and 81.0% in the RA group (*p* = 0.343). **Conclusions**: Camera port swapping during TA-RPN provided adequate visualization and perioperative outcomes comparable to those achieved with RA-RPN. This may be a practical alternative, particularly for anatomically complex posterior tumors.

## 1. Introduction

Partial nephrectomy has become the standard surgical treatment for localized renal tumors, with the aim of preserving renal function while maintaining oncological control [1]. A key challenge of nephron-sparing surgery is achieving a negative surgical margin while minimizing complications such as postoperative bleeding or delayed hemorrhage due to pseudoaneurysm or arteriovenous fistula formation [2]. Among these critical steps, renorrhaphy requires meticulous hemostasis and precise closure of the collecting system, both of which rely heavily on adequate surgical visualization and working angles [3]. Inadequate exposure during tumor excision or renorrhaphy can lead to prolonged warm ischemia time, increased blood loss, or the need for secondary interventions [4].

Surgical approaches to the kidney vary depending on the tumor location and the surgeon’s preference. The transperitoneal approach (TA) is the most commonly used route for partial nephrectomy owing to its familiar anatomical landmarks and ample working space [1]. However, TA can be challenging for posterior renal tumors, as it often requires extensive mobilization of the kidney and perinephric fat to achieve adequate exposure [5]. Excessive mobilization not only prolongs the operative time, but also increases the risk of perirenal adhesions and injury to the surrounding structures [6].

The retroperitoneal approach (RA) offers a more direct route to posterior renal lesions, often allowing quicker access and potentially reducing the need for kidney mobilization [7]. Several studies have reported favorable perioperative outcomes with RA, particularly in patients with posterior tumors [8]. Nevertheless, RA is limited by a relatively confined working space, unfamiliar anatomical landmarks for many surgeons, and a steeper learning curve than TA [9]. As a result, RA is less commonly adopted in many institutions despite its potential benefits for specific tumor locations [10].

The advent of robotic surgical platforms, such as the da Vinci system, has significantly improved the ergonomics and precision of partial nephrectomy [11]. Robotic partial nephrectomy (RPN) facilitates precise dissection and renorrhaphy with enhanced visualization through three-dimensional magnification and articulating instruments [12]. Despite these advances, optimal visualization of the posterior renal surface remains a technical challenge when TA is used for posterior tumors [11].

To address this issue, we developed a simple camera port swapping (CPS) technique for TA-RPN. When the standard port configuration fails to provide sufficient exposure of the posterior tumor surface, the camera is relocated to a lateral working port, and the robotic arms are reassigned accordingly. This maneuver allows for a better angle of approach, improved visualization, and more efficient renorrhaphy without the need for additional instruments or increased surgical costs.

The objective of this study was to evaluate the feasibility and effectiveness of TA-RPN with CPS compared to RA-RPN for posterior renal tumors. We conducted a retrospective analysis of the perioperative outcomes, complication rates, and functional outcomes to determine whether CPS can serve as a practical alternative to RA, particularly for complex posterior lesions.

## 2. Materials and Methods

### 2.1. Ethics Statement

This study was performed in accordance with all applicable laws and regulations, good clinical practices, and ethical principles outlined in the Declaration of Helsinki. The study protocol was approved by the Institutional Review Board of Yongin Severance Hospital (approval number: 9-2025-0092). The requirement for written informed consent was waived because of this study’s retrospective design.

### 2.2. Study Population

We retrospectively reviewed the medical records of patients who underwent RPN at Gangnam Severance Hospital between 1 January 2018, and 31 August 2024. All surgeries were performed by a single experienced surgeon using the da Vinci Xi robotic system. A total of 260 patients underwent RPN during the study period. Among them, 72 patients were treated with either TA-RPN (N = 42) with the CPS technique or RA-RPN (N = 30). After excluding patients with non-posterior tumors, 64 patients with posteriorly located renal tumors were included in the final analysis. Posterior tumors were defined based on preoperative computed tomography or magnetic resonance imaging (MRI), with the majority of the tumor volume located within the posterior 180° of the renal contour. Of these 64 patients, 35 underwent TA-RPN using the CPS technique and 29 underwent RA-RPN. To minimize selection bias, 1:1 propensity score matching was performed based on tumor size and body mass index (BMI), yielding 21 matched pairs (n = 42).

We restricted the analytic cohort to posterior tumors to enable a like-for-like comparison with the RA, which inherently offers a direct posterior corridor. Although TA-CPS was occasionally applied to non-posterior lesions in our broader practice, these were excluded from both the unmatched and matched analyses to avoid location-driven indication bias.

### 2.3. Port Configuration and Camera Port Swapping

**Figure 1:**
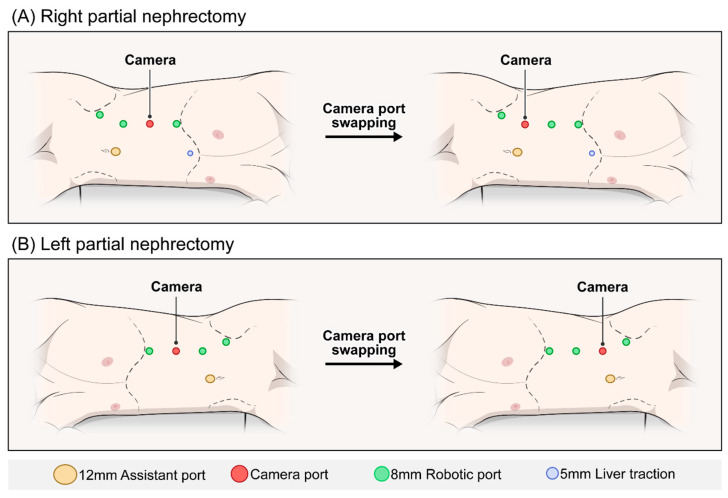
Standard and camera port-swapped configurations for transperitoneal robotic partial nephrectomy. (**A**) Right-sided partial nephrectomy: Standard port configuration (left) and camera port-swapped configuration (right). (**B**) Left-sided partial nephrectomy: Standard port configuration (left) and camera port-swapped configuration (right).

All procedures were performed using the da Vinci Xi surgical system with a four-arm robotic configuration. Patients were placed in the lateral decubitus position with the operative side up, and the operating table was slightly flexed to improve kidney exposure. Adequate padding was used to prevent pressure injury. A 12 mm assistant port was placed just superior to the umbilicus. Four 8 mm robotic ports were inserted along the midaxillary line, starting just below the costal margin, with approximately 8 cm spacing between them. The most caudal port was angled slightly toward the ipsilateral anterior superior iliac spine to optimize instrument reach. In right-sided cases, an additional 5 mm port was placed in the right upper quadrant to allow liver retraction. CPS was performed when posteriorly located tumors could not be adequately visualized using the standard port configuration. Typical intraoperative triggers included limited visualization of the tumor base, off-axis suturing angles, or instrument collision even after adequate kidney mobilization. For CPS, the camera was relocated from the central port to a more posterior lateral port, and the robotic arms were reassigned accordingly. This adjustment improved visualization of the posterior renal surface and facilitated precise tumor excision. The maneuver is reversible, and in some cases CPS was attempted and subsequently reverted to the standard configuration when ergonomics proved superior. For surgeons already proficient in the transperitoneal approach, CPS represents a straightforward adjustment rather than a separate operative technique, and thus is unlikely to entail a distinct learning curve. Figure 1 illustrates the standard and camera port-swapped configurations for the right- and left-sided TA-RPN.

### 2.4. Data Collection

Demographic and perioperative data were obtained from electronic medical records. The variables included age, sex, BMI, tumor laterality, tumor size, RENAL nephrometry score, operative time, estimated blood loss, warm ischemia time, positive surgical margin, length of hospital stay, and postoperative complications classified according to the Clavien–Dindo system. The RENAL nephrometry score was used to quantify tumor complexity, incorporating tumor size, location, and anatomical relationship to the collecting system and renal sinus [13]. The Clavien–Dindo classification categorizes surgical complications into five grades: Grade I–II (minor complications requiring no or only pharmacologic treatment), Grade III (complications requiring surgical, endoscopic, or radiologic intervention), Grade IV (life-threatening complications requiring intensive care), and Grade V (death) [14]. Renal function was evaluated by comparing estimated glomerular filtration rate (eGFR) values measured preoperatively and at final follow-up. Trifecta achievement was defined as a composite of: (1) negative surgical margin, (2) absence of postoperative complications classified as Clavien–Dindo grade III or higher, and (3) preservation of renal function, defined as a postoperative eGFR ≥ 90% of the preoperative value.

### 2.5. Statistical Analysis

All statistical analyses were performed using R software (version 4.5.1; R Foundation for Statistical Computing, Vienna, Austria). Propensity score matching was performed with the “MatchIt” package (version 4.5.0). A 1:1 nearest-neighbor matching algorithm without replacement and a caliper width of 0.2 was applied, based on tumor size and BMI. Continuous variables are expressed as medians with ranges and were compared using the Mann–Whitney U test. Categorical variables were compared using the chi-squared test or Fisher’s exact test, as appropriate. A non-inferiority analysis for trifecta achievement was conducted using predefined margins of −15%, −10%, and −5% as part of a sensitivity analysis. Statistical significance was set at *p* < 0.05.

## 3. Results

### 3.1. Baseline Characteristics

Before propensity score matching, 35 patients underwent TA-CPS and 29 underwent RA. Baseline characteristics were comparable between the groups (Table 1). The median age was slightly lower in the TA-CPS group (50 years; range, 27–78) than in the RA group (55 years; range, 42–84), although the difference was not statistically significant (*p* = 0.062). The proportion of female patients was nearly identical between the two groups (34.3% vs. 34.5%). Median BMI was similar (26.0 vs. 26.1 kg/m^2^, *p* = 0.604), as were tumor size (2.5 vs. 2.1 cm, *p* = 0.122) and RENAL nephrometry scores (median 6 in both groups, *p* = 0.625). Tumor laterality and location were also balanced, with right-sided lesions comprising 45.7% vs. 51.7%, and hilar (25.7% vs. 20.7%) and lateral (40.0% vs. 20.6%) tumors distributed without significant differences, indicating that baseline anatomical complexity was comparable between cohorts.

After 1:1 propensity score matching based on BMI and tumor size, 21 matched pairs (n = 42) were obtained. In the matched cohort, all key variables remained well balanced (Table 2). The median age was 46 years (range: 27–78 years) in the TA-CPS group and 55 years (range: 42–84 years) in the RA group (*p* = 0.053). The proportion of female patients (33.3% vs. 42.9%), median BMI (26.3 vs. 26.1 kg/m^2^, *p* = 0.554), tumor size (2.4 vs. 2.2 cm, *p* = 0.801), and RENAL scores (median 6 in both groups, *p* = 0.402) were not significantly different.

### 3.2. Perioperative Outcomes

In the matched cohort, the perioperative outcomes were comparable between the TA-CPS and RA groups (Table 2). The median operative time was 142 min (range, 97–362 min) in the TA-CPS group and 152 min (range, 102–225 min) in the RA group, with no significant difference (*p* = 0.890). The median warm ischemia time was 22 min (range, 0–44 min) for TA-CPS and 18 min (range, 7–45 min) for RA (*p* = 0.504). The estimated blood loss was similar between the groups, with both showing a median of 100 mL (*p* = 0.627). No positive surgical margins were observed in either group, confirming complete oncologic resection. No postoperative complications of Clavien–Dindo grade III or higher occurred in the TA-CPS group, while one occurred in the RA group—a pseudoaneurysm requiring angioembolization followed by full recovery (*p* = 1.000). The median length of hospital stay was 4 days in both groups (*p* = 0.989). Trifecta achievement rates were 95.2% for TA-CPS and 81.0% for RA (*p* = 0.343).

Subgroup analyses stratified by BMI (<25 vs. ≥25 kg/m^2^), RENAL score (<6 vs. ≥6), tumor size (<4 cm vs. ≥4 cm), and tumor location (hilar vs. lateral) revealed no significant differences in trifecta achievement between the two groups (Table 3).

Non-inferiority analysis further confirmed that TA-CPS was not inferior to RA across all pre-specified margins (−15%, −10%, −5%). The absolute difference in trifecta achievement was +14.3% (95% CI, −4.8% to +33.4%), with corresponding *p*-values of 0.0013, 0.0064, and 0.0239 for the −15%, −10%, and −5% non-inferiority margins, respectively (Table 4). Even under the most stringent −5% margin, TA-CPS satisfied non-inferiority criteria, and the upper bound of the confidence interval numerically favored CPS, raising the possibility of improved outcomes, though this observation requires validation in larger cohorts.

## 4. Discussion

**Figure 2:**
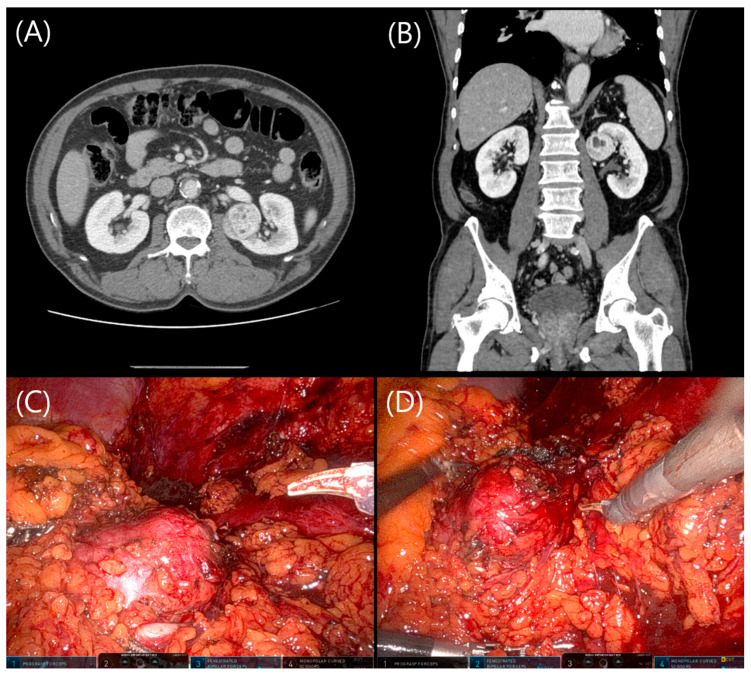
Preoperative and intraoperative imaging of a left renal tumor located at the postero-medial upper pole. (**A**,**B**) Axial and coronal views of abdominal CT show a well-defined enhancing mass at the upper pole of the left kidney, primarily located on the posterior and medial aspect. (**C**) Standard transperitoneal view during robotic partial nephrectomy demonstrates limited visualization of the posterior tumor surface. (**D**) After camera port swapping, improved visualization is achieved, allowing better exposure and instrument access for tumor excision.

Intraoperative camera repositioning is not unique to urologic surgery. For example, in robot-assisted nephroureterectomy for upper tract urothelial carcinoma, the camera is often shifted between ports to facilitate the visualization of different surgical fields [15]. Similar strategies have also been described in other robotic procedures such as total mesorectal excision and hepatic resection, where shifting the camera between ports improves the visualization of different anatomical quadrants [16]. However, these approaches often target distinct operative fields, whereas in TA-RPN, the surgical target (the kidney) remains constant. CPS adapts this concept to optimize posterior exposure within a single operative field, enhancing working angles for tumor excision and renorrhaphy without requiring additional ports or increasing costs. Although experienced surgeons may occasionally use similar adjustments as needed, our study is the first to formally define and quantitatively evaluate CPS using clinical data, establishing it as a reproducible and effective technique (Figure 2).

In the laparoscopic era, adjustments of port configuration, including camera position, were frequently required to obtain adequate exposure, a practice that clearly reflected the ergonomic limitations of rigid, non-articulating instruments and the fixed visual axis of laparoscopy [17]. In larger series of laparoscopic partial nephrectomy, posterior, hilar, and complex tumors were consistently described as technically demanding due to restricted visualization, and surgeons often relocated the camera between medial and lateral ports to optimize exposure while minimizing the number of trocars [4]. With the transition to robotic platforms, the availability of wristed instruments and enhanced three-dimensional vision substantially lowered the technical complexity compared with laparoscopic partial nephrectomy, and as a result, systematic camera repositioning became less emphasized [18]. Nevertheless, challenges remain for certain anatomically unfavorable tumors. Posterior, hilar, and deeply endophytic lesions have consistently been associated with longer ischemia times, increased operative complexity, and higher risk of positive margins [13,19]. In this context, our CPS technique represents a pragmatic return to fundamental laparoscopic principles, reintroduced into the robotic setting in a structured and reproducible manner. Rather than being viewed as a novel experiment, CPS is better understood as a natural extension of established practice, designed to restore flexibility of visualization while preserving the precision advantages of robotic surgery.

Robotic partial nephrectomy aims to optimize oncological control while preserving renal function and minimizing perioperative complications [20]. To evaluate surgical quality in RPN, the concept of “trifecta”—first introduced by Hung et al. in 2013 as negative surgical margins, no urological complications, and minimal renal functional decrease—has since evolved in subsequent studies [21]. Many series now define it more specifically as negative surgical margins, warm ischemia time ≤ 25 min, and absence of Clavien–Dindo grade III or higher complications—a benchmark widely adopted for immediate perioperative success [22]. In this study, we adopted the definition of a negative surgical margin, no major complications, and preservation of ≥ 90% eGFR to emphasize clinically relevant functional outcomes, as long-term renal function is often considered more meaningful than immediate perioperative metrics. Similar definitions have been reported in previous studies [23]. Recognizing the importance of long-term renal outcomes, more recent studies have proposed the “pentafecta” framework, which expands on the trifecta criteria by adding preservation of over 90% of eGFR and no progression of chronic kidney disease stage at 12 months [24]. While the Pentafecta framework provides a comprehensive assessment of both perioperative and long-term outcomes, we considered that its dual inclusion of eGFR preservation and chronic kidney disease stage stability was not essential for evaluating short-term outcomes. In our propensity score–matched cohort, the trifecta achievement rates in the TA-CPS and RA groups were 95.2% and 81.0%, respectively. Non-inferiority testing confirmed that TA-CPS yielded perioperative outcomes comparable to RA, supporting its feasibility and clinical effectiveness for posterior renal tumors.

The ability to achieve trifecta or pentafecta endpoints in RPN is inseparable from the quality of intraoperative exposure, since precise excision, effective hemostasis, and reliable renorrhaphy all rely on adequate visualization and working angles. Several patient-specific anatomical factors can compromise this exposure even in a standard transperitoneal setup. Perinephric fat thickness has been shown to be an independent predictor of operative complexity in RPN and is associated with longer operative time [25]. In addition to this quantitative burden, the qualitative nature of fat adhesiveness—so-called “sticky fat”—has also emerged as a strong predictor of surgical difficulty and has been validated in robotic partial nephrectomy [26]. Variations in renal anatomy such as large kidney size or atypical rotation may hinder mobilization and alter the surgical angle, while differences in thoracoabdominal configuration—such as a narrow costal margin or rigid spinal alignment—can further limit the effectiveness of table tilt and flank elevation, thereby compounding the challenge of securing an adequate working field [6]. Intraoperative exposure is further constrained by positioning factors—such as table tilt, flank elevation, and body habitus— which once the procedure is underway cannot be substantially modified without time-consuming patient repositioning or robotic re-docking [6,11]. Despite these constraints, it remains essential to secure negative margins, preserve renal function, and minimize complications—the essence of trifecta and pentafecta outcomes. In this context, CPS provides a pragmatic means to uphold these standards: by reassigning the camera to a lateral port, adequate exposure can be restored without altering patient position or docking, thereby helping to sustain surgical quality under otherwise restrictive conditions.

Although single-port robotic systems represent an evolution in minimally invasive surgery—offering smaller incisions, multiquadrant access without redocking, and improved cosmesis—they are not without limitations [27]. Comparative studies have shown that single-port approaches can be technically more challenging than multiport platforms, particularly because of reduced instrument triangulation and smaller working space, conditions that often lead to internal instrument collisions and a steeper learning curve [28]. In contrast, platforms such as the da Vinci Xi maintain full instrument dexterity and flexible port placement, with features such as boom-mounted arms, table motion, and interchangeable camera positioning among ports [29]. While single-port systems continue to evolve, the Xi platform remains versatile for procedures requiring multi-quadrant access or complex retroperitoneal dissection, where flexible port placement and dynamic visualization can be advantageous. Within this context, CPS in a familiar multi-port TA-RPN setup provides a practical and cost-effective alternative, leveraging the Xi system’s versatility to optimize intraoperative visualization—without the learning curve, added expense, or arm limitations inherent to single-port robotic systems.

Our comparative analysis demonstrated that TA-CPS achieved perioperative and functional outcomes comparable to RA, with no statistically significant differences in key variables, including operative time, warm ischemia time, blood loss, and trifecta achievement. These findings highlight CPS as a clinically viable adjunct that enables surgeons—particularly those less experienced with RA—to effectively manage posterior tumors while maintaining the advantages of the transperitoneal route. In this regard, CPS can extend the ergonomic reach of the transperitoneal approach in patients whose anatomy limits conventional strategies, including those with obesity, atypical renal orientation, or restrictive thoracoabdominal configuration, situations in which routine positional adjustments often fail to secure an adequate operative view. Moreover, CPS can be readily incorporated into existing workflows without requiring additional trocars or redocking procedures. Since this study focused on posterior renal tumors, seven patients with anterior tumors were excluded from the analysis of the 42 who underwent TA-CPS. Although TA-CPS was primarily applied to posterior tumors (83.3%), it may also be effectively used in select anterior cases, suggesting the technique is not limited to posterior lesions and can be adapted to optimize visualization when standard port configurations are inadequate. This adaptability is particularly relevant given that current guidelines do not mandate a preferred route for partial nephrectomy, and practice patterns remain heterogeneous worldwide, making CPS a practical adjunct for surgeons trained primarily in the transperitoneal approach [1]. Recent comparative analyses have emphasized that surgical approach selection remains highly institution-dependent, underscoring the importance of versatile intraoperative strategies in diverse practice settings [5]. Leveraging the Xi platform’s table motion and interchangeable camera ports, CPS functions as a brief, reversible adjustment rather than a change in operative plan, reinforcing its practicality in routine transperitoneal workflows [29]. CPS functions as a brief, reversible adjustment rather than a change in operative plan, reinforcing its practicality in routine transperitoneal workflows. Using a non-inferiority framework, we confirmed that TA-CPS was not clinically inferior to RA across all prespecified margins, and the consistent results and favorable trifecta rates support CPS as a valid alternative approach.

A key practical issue is determining the appropriate moment to employ CPS during TA-RPN. While intraoperative judgment remains the final arbiter, in our experience the maneuver was consistently prompted by recurrent scenarios—such as restricted visualization of the posterior tumor base, unfavorable suturing angles, or instrument crowding even after adequate renal mobilization. Notably, CPS is entirely reversible; in several cases the camera was shifted back to the standard port once superior ergonomics were achieved without swapping. These patterns indicate that the maneuver can be invoked on the basis of reproducible, easily recognizable intraoperative triggers, thereby enhancing its consistency, facilitating teaching to trainees, and ultimately supporting wider dissemination across different institutions and surgical settings.

A particularly important context in which CPS may provide unique benefits is multifocal renal disease, rather than solitary posterior tumors alone. Multifocal renal cell carcinoma is observed in approximately 7% of patients, yet only about 10% of these undergo partial nephrectomy, with the vast majority managed by radical nephrectomy [30]. This low utilization of partial nephrectomy largely reflects technical challenges—such as limited exposure and the difficulty of addressing both anterior and posterior lesions in a single session—rather than intrinsic oncologic inferiority, as prior studies have demonstrated comparable oncologic outcomes between nephron-sparing and radical surgery. In cases with both anterior and posterior tumors, approach selection is particularly challenging: while a retroperitoneal route offers direct access to posterior lesions, it is inherently limited for concomitant anterior excision, often requiring extensive and awkward dissection in a confined space [9]. Conversely, a transperitoneal approach affords broad anterior exposure but may struggle to provide ergonomic angles for posterior renorrhaphy [6]. CPS provides a unique compromise in such dual-location cases: surgeons may first address the anterior lesion with a standard TA configuration, and then relocate the camera to a lateral port to optimize visualization of the posterior component—all without patient repositioning or redocking. This flexibility suggests that CPS could have broader applicability to complex, multifocal disease, offering a potential alternative in situations where conventional approaches each carry intrinsic limitations.

A key strength of this study was the systematic framing and quantitative evaluation of a previously underrecognized surgical maneuver. By defining the CPS and studying its application in TA-RPN for posterior tumors, we transformed a simple adjustment into a reproducible technique with measurable outcomes. The analogy with camera port repositioning in upper tract urothelial carcinoma and other robotic procedures further supports the conceptual validity of our findings. Another notable strength lies in its pragmatic and cost-neutral nature: CPS requires no additional instruments, ports, or docking maneuvers, making it readily adoptable across institutions with established transperitoneal expertise. Because the maneuver represents a simple adjustment rather than a fundamentally new technique, it can be seamlessly integrated into established transperitoneal workflows, supporting its feasibility for adoption across different practice settings. In addition, the simplicity and reversibility of CPS confer potential educational value, as the maneuver can be readily demonstrated during standard cases and practiced without risk of additional morbidity, thereby facilitating dissemination among trainees and across institutions. Furthermore, the consistent trifecta rates across diverse subgroups in our matched analysis underscore that CPS preserved perioperative quality even in technically challenging scenarios.

Limitations include the retrospective, single-institution design and relatively small sample size despite propensity score matching, which may limit the generalizability and statistical power. In particular, this study may not have been adequately powered to detect modest differences in infrequent outcomes such as complications or renal function decline, and therefore non-significant *p* values should be interpreted with caution. Although eGFR was evaluated at the last available follow-up, providing relatively long-term data for earlier cases, the duration of follow-up was not uniform across the cohort, and long-term oncological control could not be systematically assessed. In addition, all procedures were performed by a single high-volume surgeon. While this minimizes confounding technical variability and ensures consistency in operative technique, it also limits the external validity of our findings, as outcomes may differ across surgeons and institutions with varying levels of experience. Future research should include multicenter prospective trials involving posterior and other tumor locations, with standardized follow-up to assess oncologic durability, along with assessments of learning curves and additional endpoints such as pentafecta achievement and long-term renal outcomes.

## 5. Conclusions

Camera port swapping during transperitoneal robotic partial nephrectomy offers improved visualization of posterior tumors while maintaining perioperative and functional outcomes comparable to the retroperitoneal approach. Our propensity score-matched analysis confirmed the non-inferiority of CPS with respect to trifecta achievement and other key surgical metrics. Importantly, CPS demonstrated consistent perioperative quality across subgroups, supporting its role as a broadly applicable adjunct rather than a niche maneuver. CPS is a simple, cost-neutral, and reproducible maneuver that can be readily integrated into standard robotic workflows without additional instruments or complexity. Future multicenter studies with larger sample sizes and long-term follow-up are warranted to validate the clinical applicability of CPS and to explore its utility for tumors in various anatomical locations. In addition, its potential role in challenging scenarios such as multifocal disease or anatomically unfavorable posterior lesions suggests that CPS may contribute to expanding the boundaries of partial nephrectomy while preserving the advantages of a transperitoneal approach.

## Figures and Tables

**Table 1:** Comparisons of patient characteristics and surgical outcomes between robotic partial nephrectomy using transperitoneal approach with camera port swapping technique and retroperitoneal approach.

	*n* (% or Range)		*p*-Value
	Transperitoneal Approach with Camera Port Swapping	Retroperitoneal Approach	
Number of patients	35 (54.7)	29 (45.3)	
Age, years	50 (27–78)	55 (42–84)	0.062
Sex			1.000
female	12 (34.3)	10 (34.5)	
male	23 (65.7)	19 (65.5)	
BMI, kg/m^2^	26.0 (20.3–33.8)	26.1 (20.4–36.7)	0.604
Laterality			0.802
Right	16 (45.7)	15 (51.7)	
Left	19 (54.3)	14 (48.3)	
Tumor size, cm	2.5 (0.8–6.5)	2.1 (1.0–4.6)	0.122
Hilar tumor	9 (25.7)	6 (20.7)	0.770
Lateral tumor	14 (40.0)	6 (20.6)	0.113
RENAL nephrometry score	6 (4–10)	6 (5–10)	0.625
Operative time, min	166 (97–362)	153 (102–226)	0.241
Warm ischemia time, min	23 (0–44) *	18 (7–45)	0.104
Estimated blood loss, mL	100 (0–650)	75 (0–700)	0.143
Positive surgical margin	0 (0)	0 (0)	1.000
Postoperative complication	1 (2.9)	1 (3.4)	1.000
Length of hospital stay, days	4 (3–8)	4 (3–11)	0.077
Renal function decline	5 (14.3)	4 (13.8%)	1.000
Trifecta achievement	30 (85.7)	25 (86.2)	1.000

* One case was performed using an off-clamp technique.

**Table 2:** Comparisons of patient characteristics and surgical outcomes between robotic partial nephrectomy using transperitoneal approach with camera port swapping technique and retroperitoneal approach after 1:1 propensity score matching.

	*n* (% or Range)		*p*-Value
	Transperitoneal Approach with Camera Port Swapping	Retroperitoneal Approach	
Number of patients	21 (100)	21 (100)	
Age, years	46 (27–78)	55 (42–84)	0.053
Sex			0.751
female	7 (33.3)	9 (42.9)	
male	14 (66.7)	12 (57.1)	
BMI, kg/m^2^	26.3 (20.7–30.5)	26.1 (20.4–31.3)	0.554
Laterality			0.756
Right	10 (47.6)	8 (38.1)	
Left	11 (52.4)	13 (61.9)	
Tumor size, cm	2.4 (0.8–4.5)	2.2 (1.1–4.6)	0.801
Hilar tumor	7 (33.3)	2 (9.5)	0.130
Lateral tumor	7 (33.3)	6 (28.6)	1.000
RENAL nephrometry score	6 (6–10)	6 (5–10)	0.402
Operative time, min	142 (97–362)	152 (102–225)	0.890
Warm ischemia time, min	22 (0–44) *	18 (7–45)	0.504
Estimated blood loss, mL	100 (0–400)	100 (0–700)	0.627
Positive surgical margin	0 (0)	0 (0)	1.000
Postoperative complication	0 (0)	1 (4.8)	1.000
Length of hospital stay, days	4 (3–8)	4 (3–11)	0.989
Renal function decline	1 (4.8)	4 (19)	0.343
Trifecta achievement	20 (95.2)	17 (81.0)	0.343

* One case was performed using an off-clamp technique.

**Table 3:** Subgroup comparison of trifecta achievement by surgical approach in the propensity score-matched cohort.

	Transperitoneal Approach with Camera Port Swapping		Retroperitoneal Approach	
	Trifecta Achievement/ Total (No, %)	*p*-Value	Trifecta Achievement/ Total (No, %)	*p*-Value
BMI		0.381		0.532
<25	7/8 (89.9)		5/5 (100.0)	
≥25	13/13 (100.0)		12/16 (75.0)	
RENAL score		1.000		1.000
<6	5/5 (100.0)		6/7 (85.7)	
≥6	15/16 (93.8)		11/14 (78.6)	
Tumor size		1.000		0.489
<4 cm	19/20 (95.0)		15/18 (83.3)	
≥4 cm	1/1 (100.0)		2/3 (66.7)	
Hilar tumor		0.333		1.000
No	14/14 (100.0)		15/19 (78.9)	
Yes	6/7 (85.7)		2/2 (100.0)	
Lateral tumor		1.000		1.000
No	13/14 (92.9)		12/15 (80.0)	
Yes	7/7 (100.0)		5/6 (83.3)	

**Table 4:** Non-inferiority analysis of trifecta achievement between transperitoneal approach with camera port swapping and retroperitoneal approaches in robotic partial nephrectomy (propensity score-matched cohort).

Margin	Difference	95% CI	*p*-Value	Conclusion
−15%	+14.3%	−4.8% to 33.4%	0.0013	Non-inferior
−10%	+14.3%	−4.8% to 33.4%	0.0064	Non-inferior
−5%	+14.3%	−4.8% to 33.4%	0.0239	Non-inferior

## Data Availability

The datasets generated during the current study have been anonymized and are not individually identifiable, but they are not publicly available due to institutional and ethical regulations. However, they can be made available from the corresponding author upon reasonable request.

## Abbreviations

The following abbreviations are used in this manuscript:

RPN	Robotic partial nephrectomy
TA	Transperitoneal approach
RA	Retroperitoneal approach
CPS	Camera port swapping
BMI	Body mass index
